# PSMD7 downregulation suppresses lung cancer progression by regulating the p53 pathway

**DOI:** 10.7150/jca.53613

**Published:** 2021-06-11

**Authors:** Xinchun Xu, Xiaofeng Xuan, Jieru Zhang, Hui Xu, Xiaomei Yang, Ling Zhang, Yuanjie Zhao, Hong Xu, Dawei Li

**Affiliations:** 1 Department of Ultrasound, The Affiliated Zhangjiagang Hospital of Soochow University, 68 Jiyang West Road, Suzhou, 215600, China.; 2 Department of Respiratory & Critical Care Medicine, The Affiliated Zhangjiagang Hospital of Soochow University, 68 Jiyang West Road, Suzhou, 215600, China.; 3 Department of Thoracic Surgery, The Affiliated Zhangjiagang Hospital of Soochow University, 68 Jiyang West Road, Suzhou, 215600, China.; 4 Department of Emergency, The Affiliated Zhangjiagang Hospital of Soochow University, 68 Jiyang West Road, Suzhou, 215600, China.; 5 Center for Translational Medicine, The Affiliated Zhangjiagang Hospital of Soochow University, 68 Jiyang West Road, Suzhou, 215600, China.; 6 Department of General Surgery, The Affiliated Zhangjiagang Hospital of Soochow University, 68 Jiyang West Road, Suzhou, 215600, China.

**Keywords:** deubiquitinase, PSMD7, NSCLC, LUAD, p53, cell cycle proteins

## Abstract

Lung cancer is the second most common cancer in both men and women. The deubiquitinase PSMD7, as a core component of the 26S proteasome, is critical for the degradation of ubiquitinated proteins in the proteasome. Currently, PSMD7 expression and its roles in the progression of lung cancer remain largely unknown. In this study, we assessed PSMD7 expression and investigated the underlying molecular events by which PSMD7 regulates tumor progression in non-small cell lung cancer (NSCLC). The results showed that PSMD7 is more highly expressed in NSCLC tissues than in adjacent noncancerous tissues. PSMD7 expression was also closely associated with lymph node invasion and the laterality of the tumor in lung adenocarcinoma (LUAD). A high PSMD7 level predicted poor overall survival (OS) and disease-free survival (DFS) in LUAD patients, and PSMD7 knockdown significantly reduced cell proliferation and induced G0/G1-phase cell cycle arrest, cell senescence and apoptosis. PSMD7 knockdown inhibited expression of a set of proteins regulating cell cycle progression. Depletion of PSMD7 increased p53 levels and induced p21 and puma expression in a p53-dependent manner. Importantly, knockdown of PSMD7 markedly inhibited LUAD tumor growth in a xenograft mouse model. Taken together, these findings indicate that PSMD7 may serve as a valuable prognostic indicator and potential therapeutic target in LUAD.

## Introduction

Lung cancer is the most common malignant tumor and one of the leading causes of morbidity and mortality in both China and globally 1, and the five-year survival rate is less than 20% 2. There are two main types of lung cancer: non-small cell lung cancer (NSCLC) and small cell lung cancer (SCLC) 3. NSCLC accounts for approximately 80-85% of all cases, including adenocarcinoma, squamous cell carcinoma and large cell carcinoma 3. Lung adenocarcinoma (LUAD) is the most common type of NSCLC, accounting for approximately half of all malignant lung cancers 4. The most common risk factor for NSCLC is smoking and other risks, including exposure to carcinogens and outdoor air pollution 5, 6. Currently, there are three treatment options for lung cancer: surgery, regional radiation therapy and systemic drug therapy. However, there are limited available drug treatments for patients with terminal disease 7, 8. Hence, there is an urgent need to identify key signaling pathways involved in the pathogenesis of NSCLC to discover potential therapeutic targets and improve treatment.

The ubiquitin-proteasome system plays a key role in multiple fundamental biochemical processes, including regulated degradation of proteins, cell cycle progression, apoptosis, DNA damage repair, and signal transduction 9-12. The 26S proteasome is the main mediator of protein degradation and is composed of the 20S proteasome core particle (CP) and one or two 19S regulatory particles (RPs) 13. RPs are responsible for the recognition and recruitment of poly-ubiquitylated substrates as well as their deubiquitylation, ATP hydrolysis, and insertion into the core particle 14, 15. CP can promote substrate proteolysis and protect proteolytic activity and isolate it from the environment, thus avoiding indirect damage. The proteasome is an attractive drug target for the development of cancer therapies. In fact, bortezomib, a small molecule proteasome inhibitor, has been successfully used clinically for targeting of the proteasome in refractory multiple myeloma and mantle cell myeloma 16, 17.

PSMD7 (26S proteasome non-ATPase regulatory subunit 7), also known as 26S proteasome non-ATPase subunit Rpn8, is reported to be an ATP-independent component of the 19S modulator subunit, that binds with PSMD14 to form a heterodimer as a functional component for efficient degradation of ubiquitinated substrates in the proteasome^18^. Our previous study indicated that PSMD14 is upregulated in lung adenocarcinoma and predicts a poor prognosis 19. Furthermore, we found that PSMD7 regulates cell fate and is associated with disease progression in breast cancer 20. In addition, PSMD7 can regulate the G2/M phase cell cycle transition during HIV infection 21. Knockdown of PSMD7 induces apoptosis and inhibits tumorigenesis in esophageal squamous cell carcinoma 22. However, whether PSMD7 contributes to the progression of NSCLC remains unclear.

In this study, we explored expression of PSMD7 and its prognostic significance in NSCLC. We found that elevated PSMD7 levels were associated with poor clinical stages, OS and DFS in LUAD patients. PSMD7 knockdown induced cell cycle arrest, senescence, and apoptosis and suppressed LUAD tumor growth in a xenograft mouse model. Thus, PSMD7 may be used as a prognostic indicator and a promising therapeutic target in LUAD.

## Materials and methods

### Patients

A total of 25 pairs of NSCLC specimens and corresponding adjacent noncancerous tissues were obtained from patients who underwent curative surgery at The Affiliated Zhangjiagang Hospital of Soochow University between March 2019 and February 2020. None of the patients received any previous chemotherapy or radiotherapy. Histological diagnoses of primary NSCLC were confirmed by at least two experienced histological pathologists at the hospital. All tumor stages were determined according to the American Joint Committee on Cancer guidelines (http://www.cancerstaging.org/). The tumor samples were carefully isolated during surgery, and matched noncancerous lung tissues were collected at least one centimeter away from the edge of the lesion. The tissue samples were immediately frozen and stored at -80 °C for further detection. The demographic, clinical and pathological features of all patients have been previously described 19. Written permission was requested and received from all NSCLC patients in the study. The experimental procedures were approved by The Zhangjiagang Hospital Institutional Review Board (No. 2019001).

### Bioinformatics analysis

We used the publicly available clinical information provided by cBioportal for Cancer Genomics (TCGA) databases (http://www.cbioportal.org/), which include basic demographic and clinical information as well as survival status after surgery, to assess the correlation between PSMD7 and clinical pathological status as well as patient survival among NSCLC patients. We retrieved clinical information from NSCLC patients, including 510 LUAD and 484 LUSC patients, for correlation analysis. The demographic and clinical information is shown in Supplementary Table 1 and Supplementary Table 2. The partial patients' information is missing from TCGA database as indicated by not available from both tables. We extracted data for cases with higher and lower quartiles for PSMD7 expression analysis: including 259 patients for OS and 210 patients for DFS in LUAD and 240 patients for OS and 182 patients for DFS analysis in LUSC. We also compared the expression of PSMD7 in normal lung tissues and NSCLC tissues using TCGA databases provided by UALCAN websites (http://ualcan.path.uab.edu/). The mRNA expression of PSMD7 was also retrieved from TCGA databases (http://ualcan.path.uab.edu/) for 59 normal lung tissues and 515 LUAD tissues and 52 normal lung tissues and 503 LUSC tissues, and assessed.

### Cell culture

Human LUAD cell lines H1299 and A549 were cultured in Dulbecco's Modified Eagle Medium (DMEM, Corning, USA) supplemented with 10% fetal bovine serum (FBS) and antibiotics composed of 100 U/mL penicillin and 100 µg/mL streptomycin in a humidified atmosphere at 37 °C with 5% CO_2_. When cell confluence reached 75-80%, the cells were trypsinized and passaged every 2-3 days.

### Western blotting and antibodies

Total proteins of tissues and cells were extracted using FLAG lysis buffer (50 mM Tris·HCl, pH 7.9, 137 mM NaCl, 10 mM NaF, 1 mM EDTA, 1% Triton X-100, 0.2% Sarkosyl, 10% glycerol) containing protease inhibitor cocktail (Sigma, St Louis, Missouri, USA). Cell lysates were sonicated for 10 s and then incubated on ice for 30 mins. Supernatants were separated after the cell extract was centrifuged at 12000 g at 4 °C for 30 mins Protein concentrations were measured using the BCA assay (23227, Pierce, Rockford, IL, USA). A total of 40 µg lysate was loaded onto 10% SDS/PAGE gels for electrophoresis and then transferred to nitrocellulose membranes (GE Healthcare, Munich, Germany). The membranes were blocked in 5% skimmed milk for 1 h at room temperature and then incubated overnight at 4 °C with gentle shaking with the diluted primary antibodies against the following: PSMD7 (sc-390705), cyclin D1 (sc-450), cyclin B1 (sc-7393), CDK1 (sc-54), Cdc25c (sc-13138), p53 (sc-126), p27 (sc-1641), p21 (sc-53870), Rb (sc-47562), Mdm2 (sc-965) from Santa Cruz Biotechnology (CA, USA), phospho-Rb (9308, Cell Signaling Technology, Danvers, MA, USA), CDK4 (66950-1-Ig, Proteintech, Rosemont, IL, USA), cyclin B1 (55004-1-AP, Proteintech, Rosemont, IL, USA), Caspase-3 (19677, Proteintech, Rosemont, IL, USA), Cleaved caspase-3 (9661, Cell Signaling Technology, Danvers, MA, USA), Puma (12450, Cell Signaling Technology, Danvers, MA, USA) and (A5441, Sigma). The membranes were incubated with horseradish peroxidase-labeled secondary antibodies (GE healthcare, NA935) after extensive washes, and signals were detected using an enhanced chemiluminescent kit (ECL, WBKLS0500, Millipore, Bedford, MA, USA) with a ChemiDoc XRS (Bio-Rad, CA, USA) detection system. The signals were analyzed and quantified by ImageJ software (National Institutes of Health, Bethesda, MD, USA) with β-actin as a loading control.

### Immunohistochemistry (IHC)

The frozen tissues were sectioned at -20 °C with a thickness of 8 µm and fixed with 4% paraformaldehyde for 15 mins at room temperature. The slides were washed twice in phosphate-buffered saline (PBS) containing 0.3% Triton X-100 for 10 mins each and followed by pre-incubation in 10% normal goat serum for 1 h. The sections were then washed with PBS 3 times and incubated with a primary anti-PSMD7 antibody (1:100) at 4 °C overnight; soaking in 0.3% H_2_O_2_ for 15 mins was applied to block endogenous peroxidases. A biotinylated secondary antibody was added to the sections. After washing the sections 3 times, the tissues were incubated with a solution containing streptavidin and biotin conjugated HRP (Beyotime, China) for 1 h at room temperature and subsequently stained with 3'-diaminobenzidine (DAB, Beyotime, China) for 30 mins after extensive washing. The reaction was stopped by rinsing in H_2_O for 5 mins, and the tissues were counterstained with hematoxylin, dehydrated, cleared, and mounted in neutral resin. A Leica upright microscope (DM4000B, Leica Microsystems, Heidelberg, Germany) was used to observe the IHC staining.

### siRNA transfection

Cells were seeded in 10-mm plates, and the culture medium was replaced with Opti-MEM (Gibco) 24 hours later. H1299 and A549 cells were transfected with siRNAs using Lipofectamine 2000 (Invitrogen, USA) according to the manufacturer's instructions. The siRNA sequences were as follows: siRNA duplexes targeting PSMD7 mRNA (siPSMD7-1: 5'-GCCCUAAACUACACAAGAAUU-3' and 5'-UUCUUGUGUAGUUUAGGGCUU-3'; siPSMD7-2: 5'-UGACAUUGCCAUCAACGAAUU-3' and 5'-UUCGUUGAUGGCAAUGUCAUU-3'), si-p53: 5'-GACUCCAGGUUGAAUCUACdtdt-3' and 5'-GUAGAUUACCACUGGAGUCdtdt-3'; negative control scramble sequence (5'-UUCUCCGAACGUGUCACGUTT-3' and 5'-ACGUGACACGUUCGGAGAATT-3').

### Cell proliferation and viability assay

H1299 and A549 cells were seeded in 96-well plates (1500 cells per well) in 100 µL of complete medium. The cells were cultured for 5 days after transfection, and cell proliferation assays were performed using a kit containing WST-8 (CCK-8, CK04, Dojindo, Kumamoto, Japan). A microplate reader (Thermo Fisher Scientific) was used to measure absorbance of each well at 450 nm. The results were analyzed, and the results are shown as the mean optical density (OD) ± SD. The crystal violet assay was used to evaluate the viability of H1299 and A549 cells after transfection with NC and si-PSMD7. The cells were seeded in 6-well plates at a density of 50,000 cells per well, fixed with methanol and stained with 0.1% crystal violet after 72 hours of transfection.

### Flow cytometric analysis

H1299 and A549 cells were transfected with NC and si-PSMD7 and harvested 72 hours later. Both floating and adherent cells were collected by centrifugation at 1000x g for 10 mins and fixed in 70% ice cold ethanol diluted with PBS at 4 °C overnight. The cells were washed twice in PBS, stained with PI/RNase staining buffer (BD Biosciences, San Diego, CA, USA) and incubated at 37 °C for 30 minutes. The distribution of the cell cycle was measured by flow cytometry (Beckman Coulter, Brea, CA, USA).

### Senescence-associated β-galactosidase (SA-β-Gal) assay

Senescent cells were detected using a Senescent Cells Staining Kit (C0602, Beyotime) at 72 hours after transfection according to the manufacturer's instructions. Cells stained with β-galactosidase were viewed under an inverted fluorescence microscope (DMI4000B, Leica Microsystems). Representative pictures were captured for different conditions. The SA-β-Gal-positive senescent cells were calculated, and the results are expressed as a percentage of the total number of cells in five separate fields. Two independent experiments were conducted to calculate the average value of SA-β-Gal-positive cells and the standard deviation.

### Real-time PCR (RT-PCR)

Total RNA was extracted from tissue and cell samples using TRIzol reagent (Invitrogen, Carlsbad, CA, USA) according to the manufacturer's protocol. Reverse transcription was performed using a Thermo Scientific RevertAid H Minus First-Strand cDNA Synthesis Kit (K1632, Thermo Fisher, MA, USA). RT-PCR was performed using a QuantStudio Dx Real-Time PCR Instrument (Applied Biosystems) with SYBR Green Master Mix (Applied Biosystems, CA, USA) under the following conditions: initial denaturation at 95 °C for 10 min, followed by 40 cycles of 95 °C for 15 seconds and 60 °C for 1 min. The results were analyzed by the comparative cycle threshold method, and GAPDH was used as an internal control. The sequences of primers (Sangon, Shanghai, China) were as follows: PSMD7 F: 5'-TTGGAGCAGAGGAAGCTGAG-3', R: 5'-CTGACATCTGGCAGCAGGTT-3'; cyclin D1, F: 5'-ACGAAGGTCTGCGCGTGTT-3', R: 5'-CCGCTGGCCATGAACTACCT-3'; CDK4, F: 5'-CCTGGCCAGAATCTACAGCTA-3', R: 5'-ACATCTCGAGGCCAGTCATC-3'; cyclin B1, F: 5'-AAGAGCTTTAAACTTTGGTCTGGG-3', R: 5'-CTTTGTAAGTCCTTGATTTACCATG-3'; CDK1, F: 5'- TGGATCTGAAGAAATACTTGGATTCTA-3', R: 5'- CAATCCCCTGTAGGATTTGG-3'; Cdc25C, F: 5'- GATGTCCCTAGAACTCCAGTG-3', R: 5'- AGTTATCTCCCCACTGCTAAGA-3'; Rb, F: 5'- AGGATCAGATGAAGCAGATGG-3', R: 5'- TGCATTCGTGTTCGAGTAGAAG-3'; p21, F: 5'-CCATGTGGACCTGTCACTGTCTT-3', R: 5'-CGGCCTCTTGGAGAAGATCAGCCG-3'; p27, F: 5'- TTTGACTTGCATGAAGAGAAGC-3', R: 5'- AGCTGTCTCTGAAAGGGACATT-3'; p53, F: 5'-AGTGTGGTGGTGCCCTATGAG-3', R: 5'-GCCCATGCAGGAACTGTTACA-3'; Mdm2, F: 5'-CGATGAATCTACAGGGACGCCATCG-3', R: 5'-TCCTGATCCAACCAATCACCTG-3', Puma, F: 5'-GGTCCTCAGCCCTCGCTCTC-3', R: 5'-CTTGTCTCCGCCGCTCGTAC-3', and GAPDH, F: 5'-CAGGAGGCATTGCTGATGAT-3', R: 5'-GAAGGCTGGGGCTCATTT-3'.

### Xenograft tumor growth in nude mice

A total of 9 4- to 6-week-old female nude mice (BALB/c nude mice) were purchased from Shanghai SLAC Laboratory Animal Co., Ltd. and kept in individual ventilation cages on exhaust-ventilated closed-system cage racks. All mice were housed in a temperature-controlled room (22 ± 2 °C) with 40-60% humidity and a light/dark cycle of 12 h/12 h. All animal experiments were approved by the Animal Ethics Committee of Soochow University, China. H1299 cells were transfected with NC, siPSMD7-1 and siPSMD7-2 and harvested at 24 hours after transfection. Nine mice were randomly divided into three groups with 3 mice for each group. A total of 2×10^6^ transfected tumor cells suspended in 50 µL PBS and 50 µL Matrigel matrix (356234, Corning, Glendale, AZ, USA) was subcutaneously injected into both sides of the flanks of each mouse. Subcutaneous xenograft progression was measured with Vernier calipers at days 39, 42 and 45. Tumor volume was calculated by the formula V =π× L × W^2^/6, where L represents the longest dimension and W the shortest dimension of the tumor 23.

### Statistical analysis

Statistical analysis was performed using ImageJ (version 1.8.0_112), GraphPad Instat software (GraphPad Prism 5.01, GraphPad Software Inc., San Diego, CA, USA) and SPSS software (version 19.0, Chicago, IL). Quantitative data are presented as mean values ± standard deviation (SD). The results were calculated using Student's t-test for comparison of two groups and one-way ANOVA for multiple groups. Chi-squared or Fisher's exact tests were used to analyze the correlation between PSMD7 and demographic and clinical features. Survival curves were evaluated with the Kaplan-Meier method (log-rank test). A p value <0.05 was considered statistically significant.

## Results

### PSMD7 expression is upregulated in human NSCLC tissues

**Figure 1:**
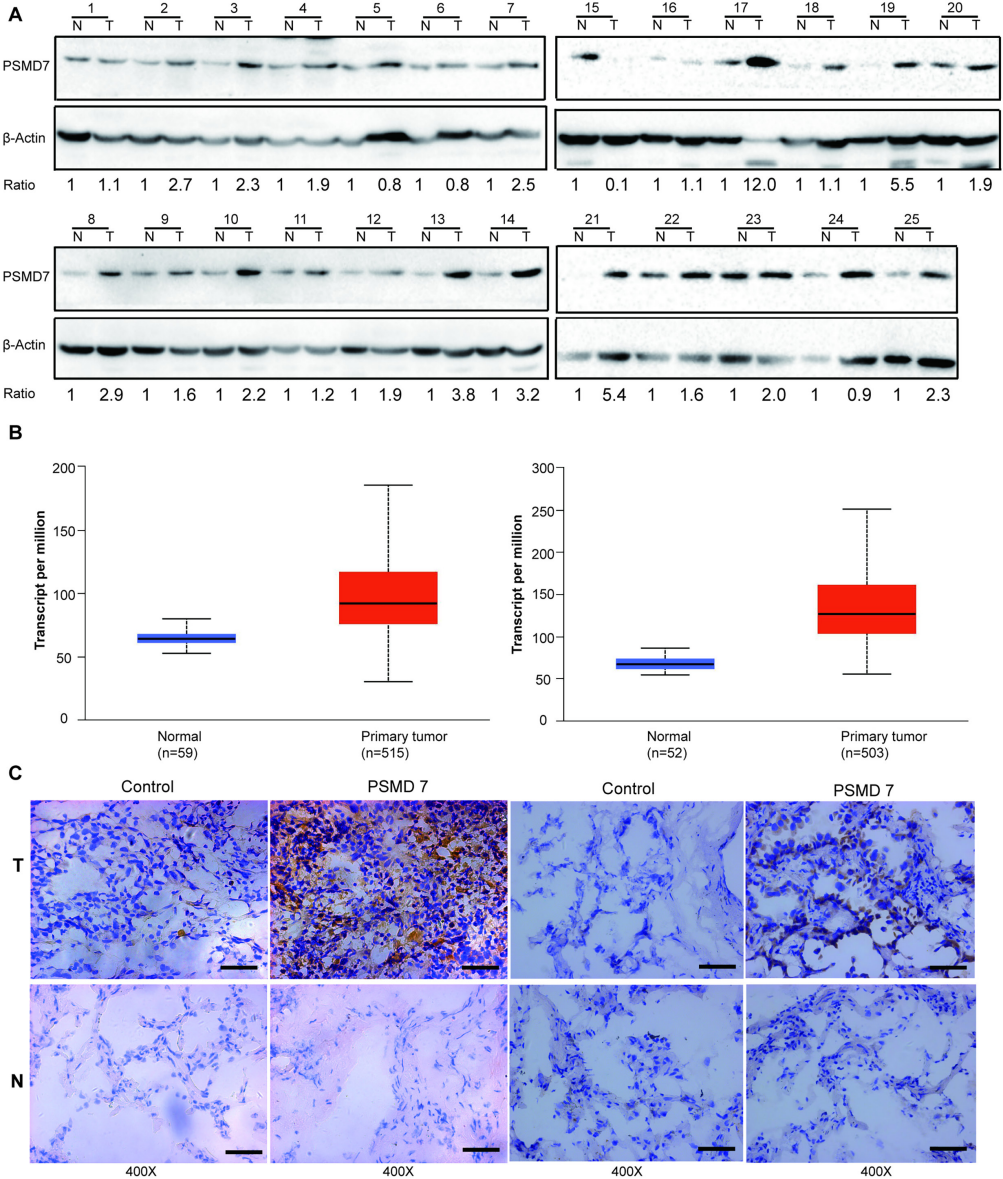
PSMD7 expression is upregulated in NSCLC tissues. **(A)** PSMD7 protein expression in 25 paired tumor (T) tissues and adjacent noncancerous (N) tissues from NSCLC patients was examined by Western blotting. β-actin was used as an internal control. **(B)** Box (25-75^th^ percentiles) and whisker (minimum-maximum) plots for PSMD7 expression in controls and NSCLC patients; the horizontal line inside the box indicates the median (the 50^th^ percentile). P-values were calculated by the Kruskal-Wallis test. **(C)** Typical IHC staining of PSMD7 in paired tumor (T) and adjacent noncancerous (N) tissues from two representative patients. The scale bar represents 100 µm. NSCLC: non-small cell lung cancer.

PSMD14, a key functional part of the 26S proteasome, is overexpressed in most cancers 24. PSMD7 functions in a complex with PSMD14 to regulate ubiquitinated substrate degradation. To explore expression of PSMD7 in NSCLC tissues, PSMD7 protein expression was assessed in paired tumor and adjacent normal tissues from 25 NSCLC patients (Figure 1A). The results showed that PSMD7 expression was upregulated in 21 patients but downregulated in only 4. PSMD7 levels were significantly increased in NSCLC tissues compared with noncancerous tissues (p<0.001 from Fisher's exact test). Next, we retrieved PSMD7 mRNA expression information from TCGA and compared mRNA expression in normal lung and NSCLC tissues; mRNA levels of PSMD7 were evaluated in 59 normal tissues and 515 LUAD tissues and 52 normal tissues and 503 LUSC tissues. PSMD7 mRNA levels were markedly increased in LUAD and LUSC tissues compared with normal lung tissues (Figure 1B). Finally, we performed IHC staining and found PSMD7 mainly in the cytoplasm in normal tissues, whereas PSMD7 staining was detected in both the cytoplasm and nuclei in tumor tissues. Moreover, PSMD7 staining was much stronger in NSCLC tissues than in non-cancerous lung tissues (Figure 1C).

### PSMD7 expression is associated with clinical features and prognosis in LUAD patients

**Figure 2:**
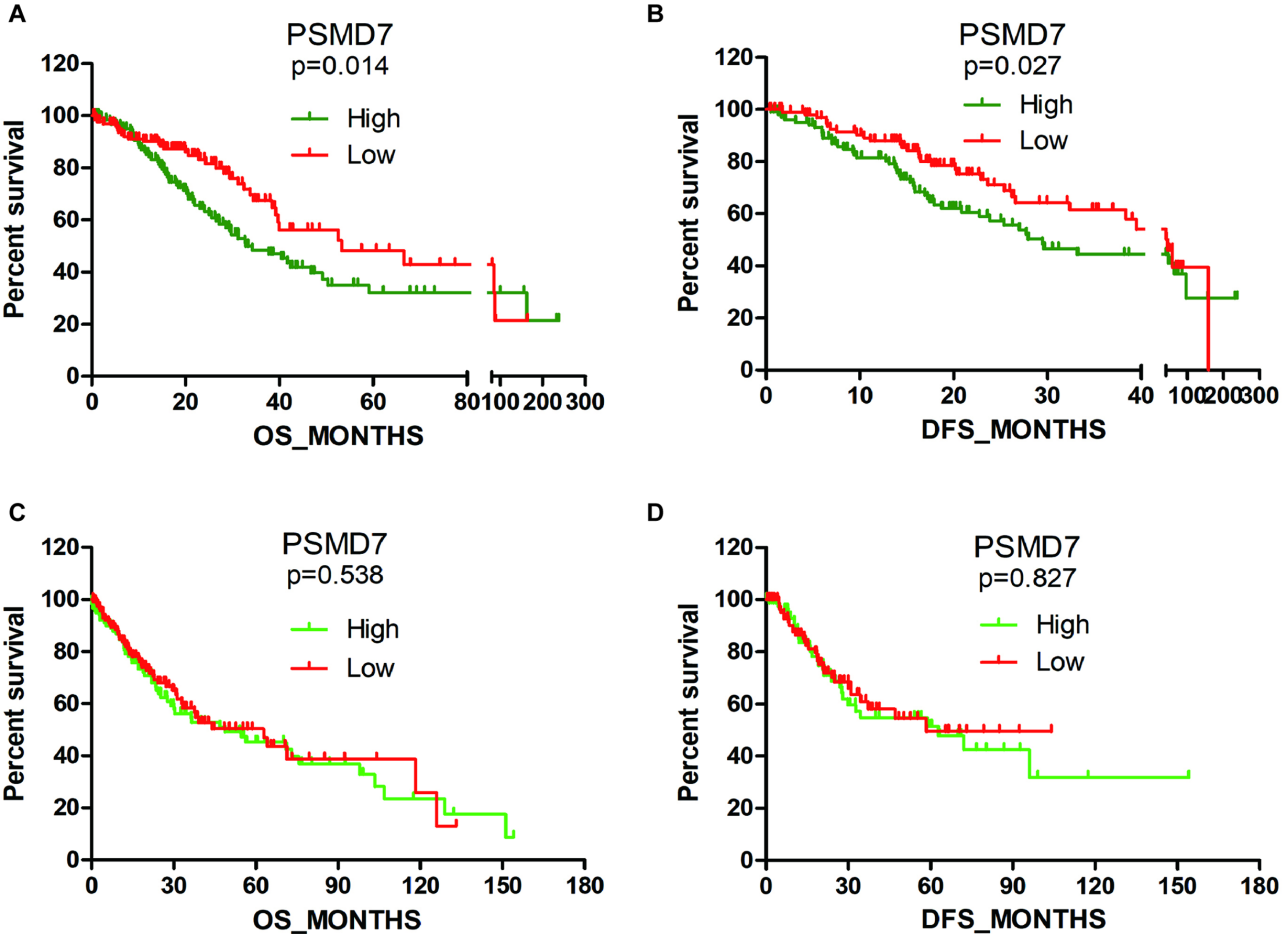
High PSMD7 expression indicates poor OS and DFS in LUAD patients. Kaplan-Meier survival analyses were conducted to evaluate the prognostic significance of PSMD7 for OS and DFS in NSCLC patients. Patient information was retrieved from the cBioportal for Cancer Genomics (TCGA) databases and analyzed by a log-rank test. NSCLC, non-small cell lung cancer; LUAD, lung adenocarcinoma; LUSC, lung squamous cell carcinoma; OS, overall survival; DFS, disease-free survival.

Subsequently, we searched TCGA to analyze the association between PSMD7 expression and clinical features. Statistical analysis revealed that PSMD7 expression was significantly associated with the laterality of the tumor and lymph node invasion in LUAD patients. Although the PSMD7 level was positively associated with sex, there was no significant correlation between PSMD7 expression and clinical and demographic features in LUSC patients (Table 1). Kaplan-Meier survival analysis was used to determine the prognostic impact of PSMD7 in NSCLC patients. The results showed that LUAD patients with lower PSMD7 expression had a significantly longer OS and DFS than the patients with higher expression (Figure 2). The median months of OS and DFS were 32.82 and 29.5 for patients with a higher level of PSMD7 expression and 53.29 and 45.27 for patients with a lower level of PSMD7 expression, respectively. Additionally, a higher PSMD7 level predicted a poor OS (HR=1.601, 95% CI=1.065-2.407) and DFS (HR=1.433, 95% CI=0.9201-2.232) in LUAD patients. Conversely, PSMD7 levels did not correlate with prognosis in LUSC patients (Figure 2), suggesting that PSMD7 is a specific prognostic marker for LUAD.

### PSMD7 knockdown induces cell cycle arrest, senescence and apoptosis in LUAD cells

**Figure 3:**
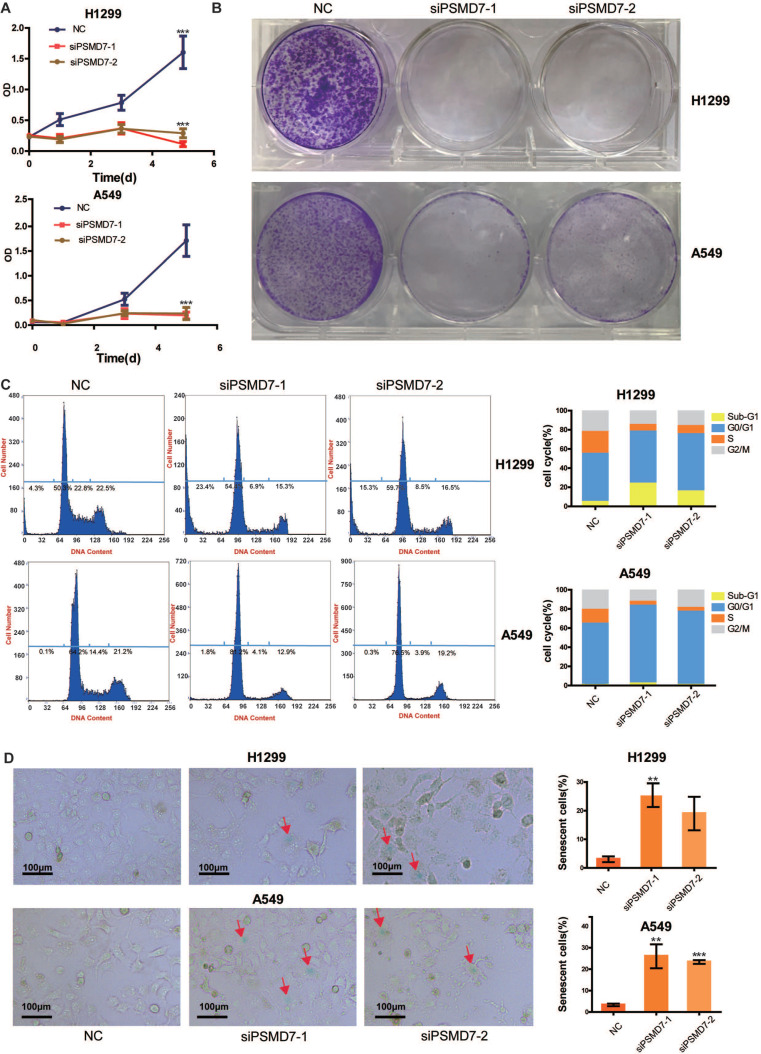
PSMD7 knockdown induces cell cycle arrest, senescence and apoptosis in LUAD cells. **(A)** The viability of H1299 and A549 cells transfected with NC, siPSMD7-1, and siPSMD7-2 was assessed by CCK-8 assays. The OD value was determined at 1 day, 3 days, and 5 days post-transfection. The assays were performed at four replicates for each group. The experiments were repeated twice, and a representative result is shown. Error bars represent the mean± SD. **(B)** Crystal violet staining was conducted to assess the survival of H1299 and A549 cells at 72 h after transfection with NC, siPSMD7-1, and siPSMD7-2. The experiments were repeated twice, and representative staining is shown. **(C)** H1299 and A549 cells transfected with NC, siPSMD7-1, and siPSMD7-2 were stained with PI and analyzed by flow cytometry. The percentages of the respective cells in each cell cycle phase at 72 h are shown. The experiments were repeated twice, and a representative result is shown. **(D)** Exponentially growing H1299 and A549 cells were transfected with NC, siPSMD7-1, and siPSMD7-2. After 72 h, the cells were fixed and incubated with SA-β-Gal overnight. The senescence-like phenotype was imaged by microscopy (10x). The number of SA-β-Gal-positive cells was counted, and the result is expressed as a percentage of the total number of cells in five separate fields. The means and standard deviation of SA-β-Gal-positive cells were derived from two independent experiments. All ***<0.001, **<0.01, *P<0.05 using unpaired Student's t test. NC, scrambled control siRNA; PI, propiodium iodide; SA-β-Gal, senescence-associated β-galactosidase.

A previous study 22 demonstrated that downregulation of PSMD7 led to decreased cell proliferation and increased apoptosis in esophageal squamous cell carcinoma. Our previous study found that PSMD7 regulates cell fate and is associated with disease progression in breast cancer 20. To investigate the potential role of PSMD7 in LUAD progression, we knocked down expression of PSMD7 using two siRNAs in H1299 and A549 cells, and cell proliferation was examined by CCK-8 assays at 1, 3, and 5 days after siRNA transfection. Knockdown of PSMD7 resulted in significant inhibition of cell proliferation in both cell lines (Figure 3A). Consistently, PSMD7-knockdown cells formed significantly fewer colonies compared to the controls, as based on crystal staining (Figure 3B). Next, the effect of PSMD7 knockdown on the cell cycle was investigated by PI staining followed by flow cytometric analysis. Our results showed that knockdown of PSMD7 induced G0/G1-phase arrest in both H1299 and A549 cells. PSMD7 knockdown also led to an increase in apoptosis (sub-G1 peak) in H1299 cells but not in A549 cells (Figure 3C). Finally, we detected cell senescence via SA-β-Gal staining after PSMD7 knockdown by staining both LUAD cell lines overnight with SA-β-Gal at 72 h after transfection. We found that the cell morphology changed from a spindle shape to an enlarged, flattened, and irregular shape after PSMD7 was knocked down in both cell lines; SA-β-Gal-stained cells were markedly increased among the PSMD7-knockdown cells (Figure 3D). In conclusion, PSMD7 knockdown induces cell cycle arrest, senescence and apoptosis in LUAD cells.

### PSMD7 knockdown regulates expression of cell cycle proteins

**Figure 4:**
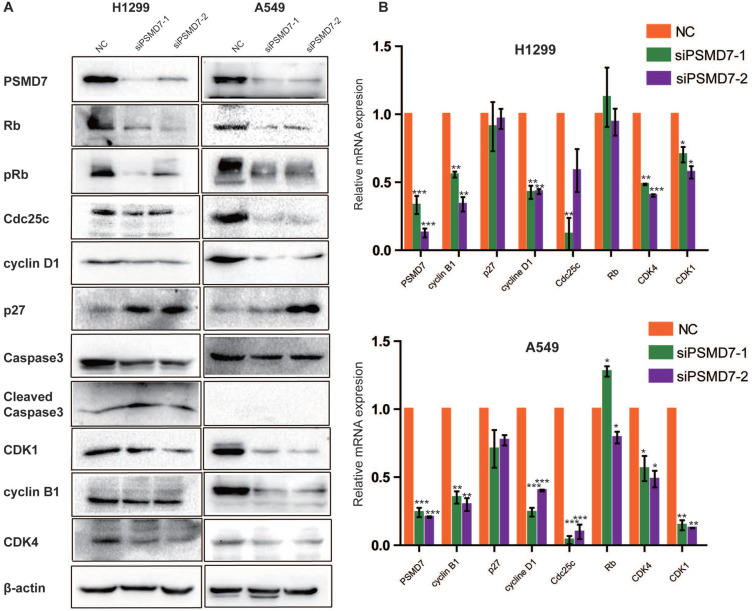
** (A)** Whole-cell lysates of H1299 and A549 cells were extracted after the cells were transfected with NC, siPSMD7-1, and siPSMD7-2 for 72 h. Western blot analysis was conducted using the indicated antibodies. **(B)** H1299 and A549 cells were transfected with NC, siPSMD7-1, and siPSMD7-2 for 72 h. mRNA expression of cell cycle proteins was detected by RT-PCR. Error bars represent the mean± SD derived from three independent experiments. β-actin was used as an internal control. NC, scrambled control siRNA.

**Figure 5:**
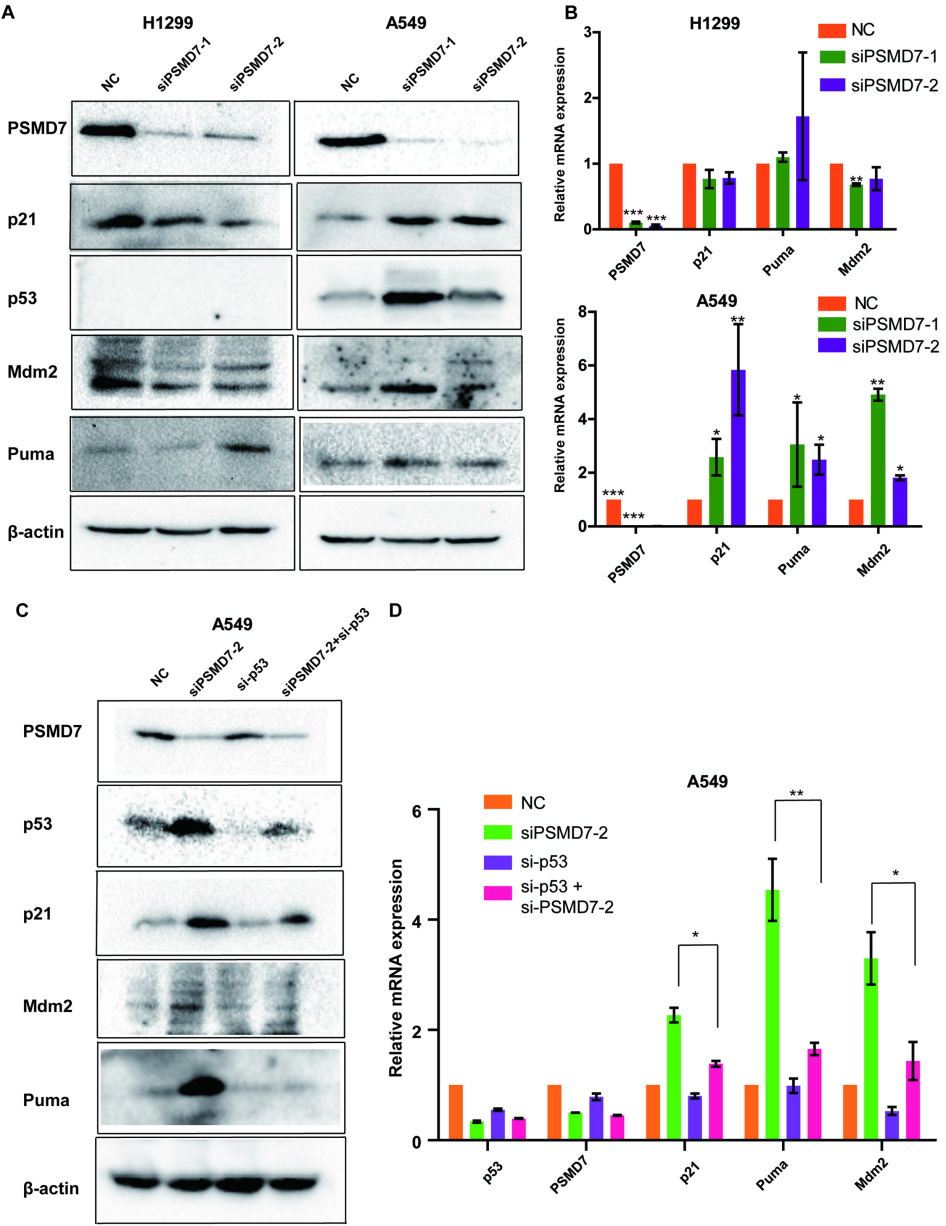
PSMD7 knockdown activates the p53 pathway in LUAD cells. **(A)** PSMD7, p53, Mdm2, p21 and Puma expression in H1299 and A549 cells was determined after PSMD7 was knocked down for 72 hours by two siRNAs. **(B)** The mRNA expression of PSMD7, p53, Mdm2, p21 and Puma was detected by RT-PCR. **(C)** PSMD7 and p53 siRNAs were co-transfected into A549 cells for 48 hours, and expression of PSMD7, p53, Mdm2, p21 and Puma was examined by Western blot analysis. (D) mRNA expression of PSMD7, p53, Mdm2, p21 and Puma was detected by RT-PCR. All **<0.01, *P<0.05 using unpaired Student's t test.

To investigate the molecular event by which PSMD7 regulates the cell cycle, apoptosis, and senescence, we examined cyclin D1, cyclin B1, CDK4, CDK1, Cdc25c, p27, Rb, pRb, Caspase-3, and Cleaved caspase-3 expression in our comparative study. According to the results, expression of cyclin D1, CDK4, CDK1, and Cdc25c was significantly reduced in both H1299 and A549 cells (Figure 4A). P27 was increased in H1299 cells but remained steady in A549 cells (Figure 5A). Additionally, levels of Rb and phosphorylated Rb (pRb) showed a significant reduction in both cell lines (Figure 4A). We then validated the observed differential expression at the mRNA level by RT-PCR analysis, and the results indicated that the levels of cyclin B1, cyclin D1, Cdc25c, CDK4, and CDK1 were significantly reduced in both cell lines at 2 days after transfection with PSMD7 siRNA (Figure 4B), suggesting that PSMD7 may play a role in transcriptional repression (Figure 4A and B). Although the mRNA level of p27 remained almost unchanged, protein expression increased in H1299 cells, suggesting posttranscriptional stabilization of the p27 protein after PSMD7 depletion.

### PSMD7 knockdown inhibits tumor growth by activating the p53 pathway

Recent studies have demonstrated that p53 is involved in PSMD14-mediated cell proliferation; p53 is upregulated in PSMD14-knockdown cells, and knockdown of PSMD14 induces cancer cell apoptosis mediated via p53 25. PSMD7 functions with PSMD14 to remove attached ubiquitin chains responsible for substrate deubiquitination during proteasome degradation. We propose that disruption of the PSMD7 and PSMD14 complex may block p53 degradation in the proteasome and therefore trigger cell cycle arrest, senescence and apoptosis in LUAD cells. To test this hypothesis, we examine the p53 pathway following PSMD7 knockdown for 72 hours. Our results showed that p53, Mdm2, p21 and Puma protein levels increased after PSMD7 knockdown in p53 wild-type A549 cells but remained steady or slightly decreased in p53-null H1299 cells (Figure 5A), suggesting that PSMD7 depletion leads to the accumulation and activation of p53. To verify whether knockdown of PSMD7 affects p53 transcriptional activity, mRNA expression of these proteins was determined after PSMD7 was knocked down in both cell lines. We found that mRNA levels of Mdm2, p21 and Puma were increased in A549 cells but not in H1299 cells (Figure 5B). To further validate the p53 role of transcriptional regulation, we knocked down both PSMD7 and p53 in A549 cells and assessed expression of p53 pathway components and found that PSMD7 and p53-double knockdown cells had markedly lower protein and mRNA levels of p21, Mdm2, and Puma than PSMD7-knockdown cells (Figure 5C and D), suggesting that the effects of PSMD7 knockdown on these proteins are dependent on intact p53 expression. Thus, PSMD7 may regulate the growth and death of LUAD cells by activating the p53 pathway.

### PSMD7 knockdown inhibited tumor growth *in vivo*

**Figure 6:**
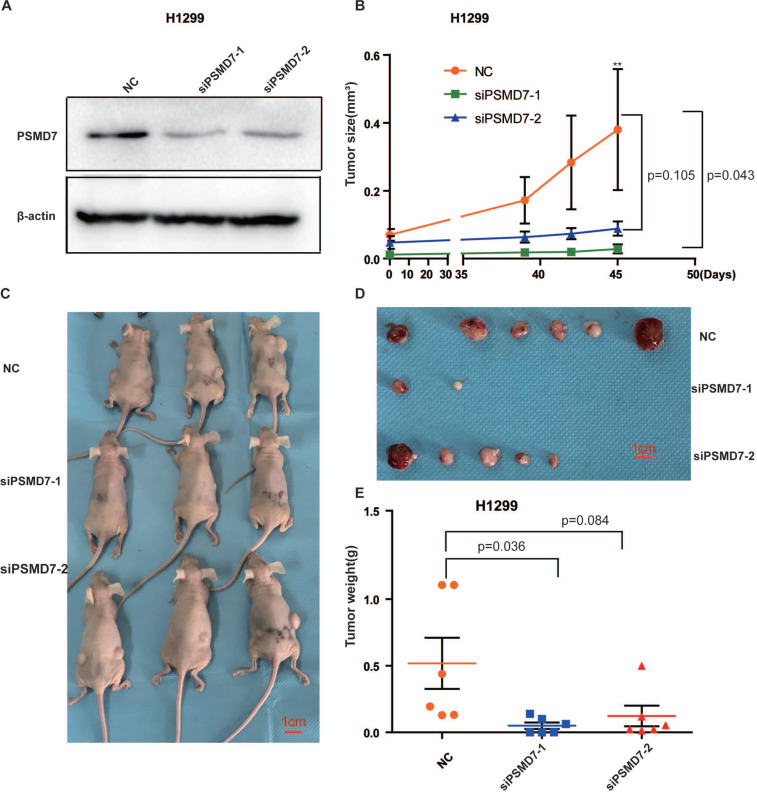
PSMD7 knockdown inhibits tumor growth *in vivo*. H1299 cells were transfected with NC, siPSMD7-1 and siPSMD7-2 for 24 hours and implanted subcutaneously with Matrigel into nude mice (n=3). **(A)** The efficiencies of PSMD7 knockdown were determined by Western blotting after transfection for 24 hours. β-actin was used as an internal control. NC, scrambled control siRNA. **(B)** Tumor volumes were measured with digital calipers at day 39, 42 and 45. **(C)** Nude mice were sacrificed at day 45. Images of the xenograft mice with tumors are shown. **(D)** The xenograft tumors were dissected from the mice; all tumors are shown. **(E)** The weight of xenograft tumors was measured. Error bars represent the mean± SD.

To assess the effect of PSMD7 on tumor growth *in vivo*, control and PSMD7-knockdown H1299 cells were subcutaneously injected into nude mice, and the knockdown efficiencies were validated by Western blotting at 24 hours after transfection. PSMD7 expression was largely inhibited by both siRNAs in these cells (Figure 6A). Tumor volumes were measured with Vernier calipers at days 39, 42 and 45. The mice were euthanized, and the tumors were removed for further measurements on day 45. Our results showed that the growth rate of tumors in the PSMD7-knockdown groups was significantly slower than that in the control group (Figure 6B, 6C and 6D). Furthermore, tumor weight was markedly lighter in the PSMD7-knockdown groups than in the control group (Figure 6E). These data suggested that PSMD7 reduction may delay tumor growth *in vivo*.

## Discussion

Lung cancer is one of the most common cancers in the world. The prognosis is the worst of all tumor types, with a five-year survival rate of 4%-17% depending on stage and regional differences 26. Thus, identifying and evaluating new biomarkers and therapeutic targets is urgent. We found that PSMD7 was elevated in NSCLC tissues. High PSMD7 expression was closely associated with lymph node invasion and predicted poor OS and DFS in LUAD patients. Thus, PSMD7 may serve as a potential prognostic predictor for LUAD patients. Furthermore, knockdown of PSMD7 reduced proliferation and induced cell cycle arrest, senescence and apoptosis by regulating cell cycle proteins and the p53 pathway in tumor cells. Knockdown of PSMD7 inhibited tumor growth in a xenograft mouse model. These findings indicate that PSMD7 is a promising drug target for LUAD treatment.

Recently, PSMD14 has been reported as an anti-proteasome target for tumor therapies in a number of cancer types, including HCC, breast cancer and ovarian cancer 27-29. Our previous study found that PSMD14 is upregulated and predicts a poor prognosis in LUAD 19. PSMD7 and PSMD14 are the core components of the 26S proteasome and are functionally closely connected. They form a dimer to activate proteasome activity and regulate ubiquitinated substrate degradation. PSMD14 knockdown induces cell cycle arrest, senescence and apoptosis by regulating cell cycle proteins in H1299 cells 30. In the current study, we assessed PSMD7 expression and its prognostic significance in NSCLC and observed that the expression of cell cycle proteins cyclin D1, CDK4, CDK1, and Cdc25c was markedly reduced in PSMD7-knockdown cells, suggesting that PSMD7 regulates gene transcription directly or indirectly. Consistently, our recent report indicated that PSMD7 regulates cell fate and disease progression in breast cancer 20. Therefore, PSMD7 functions in a variety of tumor types, and blocking it may be a promising strategy to inhibit tumor growth. Our findings of elevated proteasome components in tumor tissues support the notion that oncogenic addiction to high 26S proteasome activity is a common characteristic for many tumor types 31.

p53 is activated in response to various oncogenic stresses and behaves as a tumor suppressor via canonical tumor suppressive functions, including regulating the cell cycle, senescence and apoptosis, and non-canonical functions, such as metabolic stress modulation 32, 33. Recent studies have demonstrated that p53 is stabilized in PSMD14-knockdown cells and that depletion of PSMD14 may induce cancer cell apoptosis mediated by p53 activation 19. Our data show that PSMD7 can also increase p53 protein levels and activate transcription of Mdm2, p21 and Puma in a p53-dependent manner. Disruption of PSMD14 and PSMD7 heterodimers may affect p53 degradation in the proteasome. Our findings raise the possibility that inducing p53 activity by inhibiting proteasome activity may suppress tumor growth. Bortezomib, a proteasome inhibitor, has been demonstrated to cause apoptosis in solid tumors 29. Previous studies have shown that bortezomib induces apoptotic cell death in either a p53-dependent or p53-independent manner 32, 33. Notably, targeting PSMD7 may inhibit tumor growth in both p53 wild-type and mutant tumor types.

## Conclusions

In summary, our results show that the PSMD7 level is increased in lung cancer tissues and is closely related to clinical features. A higher PSMD7 level is also associated with unfavorable clinical outcomes in LUAD patients. Knockdown of PSMD7 induces cell cycle arrest, senescence, and apoptosis by regulating cell cycle proteins and the p53 pathway in lung cancer cells. Our results suggest that PSMD7 is a potential prognostic indicator and therapeutic target for lung cancer.

## Supplementary Material

Supplementary table S1.

Supplementary table S2.

## Figures and Tables

**Table 1:** Expression of PSMD7 correlates with demographic and clinical pathological characteristics in NSCLC (LUAD and LUSC) patients

	LUAD PSMD7		*p* value	LUSC PSMD7		*p* value
	high	low		high	low	
**Age**						
≥60	171	184	0.190	194	194	1.000
<60	75	61		43	44	
**Sex**						
Male	120	116	0.790	189	169	0.049*
Female	135	139		53	73	
**Tobacco-smoking history**						
Stage3-5	150	154	0.782	166	161	0.691
Stage1-2	98	94		71	76	
**Prior malignancy history**						
Negative	214	210	0.723	212	218	0.471
Positive	41	45		29	24	
**Laterality**						
Left	112	87	0.022*	105	100	0.638
Right	135	161		122	128	
**Lung parenchyma location**						
Peripheral lung	68	57	0.122	49	42	0.421
Central lung	26	37		68	75	
**Residual tumors**						
Negative (R0)	167	174	0.134	191	195	0.445
Positive (R1/R2)	11	5		10	6	
**Tumor size**						
T1	80	87	0.571	54	54	1.000
T2-T4	173	167		188	188	
**Lymph node invasion**						
Negative	150	178	0.011*	150	157	0.567
Positive	99	72		89	82	
**Distant metastasis**						
Negative	170	171	1.000	194	200	0.068
Positive	13	12		6	1	
**Tumor TNM Stage**						
I/II	189	204	0.129	189	202	0.159
III/IV	62	47		51	38	

Clinical information for NSCLC patients (510 LUAD and 484 LUSC patients) was retrieved from cBioportal for Cancer Genomics (TCGA) databases for correlation analysis. We analyzed 510 LUAD patients with complete information for sex and prior malignancy history. Age is missing from 19 LUAD patients; tobacco-smoking history from 14 LUAD patients; laterality from 15 LUAD patients; location of lung parenchyma from 322 LUAD patients; residual tumors from 47 LUAD patients; tumor size from 3 LUAD patients; lymph node invasion from 11 LUAD patients; distant metastasis from 56 LUAD patients and tumor TNM stages from 8 LUAD patients. We analyzed 484 LUSC patients with complete information for sex and tumor size. Age is missing from 9 LUSC patients; tobacco-smoking history from 10 LUSC patients; prior malignancy history from 1 LUSC patient; laterality from 29 LUSC patients; location of lung parenchyma from 250 LUSC patients; residual tumors from 82 LUSC patients; lymph node invasion from 6 LUSC patients; distant metastasis from 83 LUSC patients and tumor TNM stages from 4 LUSC patients. NSCLC: non-small cell lung carcinoma; LUAD: lung adenocarcinoma; LUSC: lung squamous cell carcinoma. *p<0.05 was considered significant.

## Abbreviations

PSMD7: 26S proteasome non-ATPase regulatory subunit 7
NSCLC: non-small cell lung cancer
LUAD: lung adenocarcinoma
LUSC: lung squamous cell carcinoma
OS: overall survival
DFS: disease-free survival
GEO: Gene Expression Omnibus
TCGA: The Cancer Genome Atlas
DMEM: Dulbecco's modified Eagle's medium
FBS: fetal bovine serum
RT-PCR: real-time PCR
ECL: enhanced chemiluminescence
IHC: immunohistochemistry
PBS: phosphate buffered saline
